# Seizures Associated With High-Dose Cefazolin in a Patient With Renal Dysfunction: A Case Report

**DOI:** 10.1177/08971900251326735

**Published:** 2025-03-12

**Authors:** Kulwant Kingra, Robert E. Ariano, Aditya Sharma

**Affiliations:** 1 Max Rady College of Medicine, Rady Faculty of Health Sciences, University of Manitoba, Winnipeg, MB, Canada; 2 Department of Internal Medicine, University of Manitoba, Winnipeg, MB, Canada; 3 St. Boniface Hospital Pharmacy, Winnipeg, MB, Canada; 4 College of Pharmacy, Rady Faculty of Health Sciences, University of Manitoba, Winnipeg, MB, Canada

**Keywords:** cefazolin, seizures, renal dysfunction, neurotoxicity

## Abstract

**Introduction and Objective:** Cefazolin-induced encephalopathy and seizures are possibly related to excessive dosing; especially in those with renal dysfunction. This report aims to highlight the importance of dose adjustments of cefazolin in patients with diminished renal function. **Case Presentation:** An 87-year-old female with a history of cognitive impairment, remote cerebellar infarcts, hypertension, and hypothyroidism presented with acute delirium associated with a urinary tract infection. Her condition worsened and she was found to have a methicillin-sensitive *Staphylococcus aureus* bacteremia for which she was started on cefazolin 2 grams intravenously every 4 hours. Based on her renal function, recommended dosing would have been 2 grams intravenously every 12 hours. After 3 days on this regimen her mentation declined and she suffered a tonic-clonic seizure. She did not regain consciousness and was transitioned to comfort care prior to her death. **Discussion:** Supratherapeutic dosing of cefazolin may have led to significant neurotoxic effects. Neurotoxicity and seizures can occur with drug accumulation from an increase in excitatory neurotransmitters along with a decrease in inhibitory neurotransmitter activity. The effect is potentiated by older age, pre-existing central nervous system conditions, and renal failure. Therapeutic drug monitoring is a potential strategy to limit the risk of drug toxicity. **Conclusion:** This case outlines a poor outcome in the context of high-dose cefazolin. It serves as a reminder to clinicians for ongoing pharmacovigilance in adhering to treatment guidelines.

## Introduction

Cefazolin is an intravenous beta-lactam antibiotic within the cephalosporin class, and is widely used in the treatment of various infections. The typical starting dose of cefazolin for most infections, including methicillin-susceptible *Staphylococcus aureus* (MSSA) bacteremia, is 2 grams (g) intravenously (i.v.) every 8 hours; with recommended dose reductions to 0.5-1 g i.v. every 12 or 24 hours depending on the level of renal function.^1^

Beta-lactam antibiotics, specifically cephalosporins, have been associated in the literature with central nervous system (CNS) adverse drug reactions (ADRs) which would include both confusion and seizures.^2^ Lacroix et al performed an analysis of over 500 cephalosporin related serious CNS ADRs from the French Pharmacovigilance database from 1987 to 2017 and found the most involved antibiotics being cefepime (33.1%), ceftriaxone (29.7%), ceftazidime (19.6%), cefotaxime (9%), and cefazolin (2.9%).^2^ They found that the mean time of onset for CNS ADRs was just under 8 days, with a majority of cases having elevated cephalosporin plasma levels in the context of renal impairment.^2^ The proposed mechanism behind CNS toxicity is related to an increase in glutamatergic excitation with a decrease in inhibitory neurotransmitter activity.^1^ Overall, there is a sparsity in the literature exploring the neurotoxic effects of intravenous cefazolin in humans. The following case outlines the use of high-dose cefazolin for a patient with MSSA bacteremia and concurrent kidney dysfunction, ultimately resulting in a poor outcome. This report aims to highlight the significance of appropriately dosing antibiotics in patients with diminished renal function to reduce the risk of major adverse effects.

## Case Presentation

An 87-year-old female with a medical history of cognitive impairment, remote cerebellar infarcts, hypertension, and hypothyroidism, was transferred from a personal care home to hospital with acute delirium. She was seen in the emergency room department and found to be alert and oriented to name and month with a grossly normal neurologic exam. No other concerning features were identified on physical exam. Initial investigations demonstrated a white blood cell (WBC) count of 13.6 × 10^9^/L with neutrophilic predominance, hemoglobin of 11.1 g/dL, creatinine of 1.64 mg/dL, and a leukocyte esterase and nitrite positive urine specimen. Her baseline creatinine was noted to be around 1.5 mg/dL. Her other electrolytes, including a sodium of 135 mEq/L and corrected calcium of 9.1 mg/dL, were within normal limits. She was started on nitrofurantoin for a suspected urinary tract infection. Unfortunately, a urine culture was not performed prior to starting antibiotics. No other focus of delirium was evident with a clear chest radiograph and no acute intracranial abnormality on computed tomography (CT) scan of the brain. On day 2 of presenting to hospital she was transferred to a lower acuity care community hospital given her hemodynamic stability.

On day 6 of hospitalization, she developed a fever, tachycardia, cough, and shortness of breath, with a WBC count rise to 29.4 × 10^9^/L and creatinine increase to 1.92 mg/dL. A chest radiograph showed patchy bilateral infiltrates, and blood cultures were drawn prior to being started on broad-spectrum coverage with appropriately dosed piperacillin/tazobactam. She remained on room air and did not require oxygen supplementation. On day 8, blood cultures came back positive for an MSSA bacteremia, and her antibiotics were narrowed to cefazolin. Her creatinine at this time had risen to 2.38 mg/dL and she was started on cefazolin at 2 grams i.v. every 4 hours. In retrospect, a dosage regimen of 2 grams i.v. every 12 hours was more appropriate, given her creatinine clearance of around 16 mL/min/1.73 m^2^ (Cockcroft-Gault equation).^1,3^

**Figure 1.**
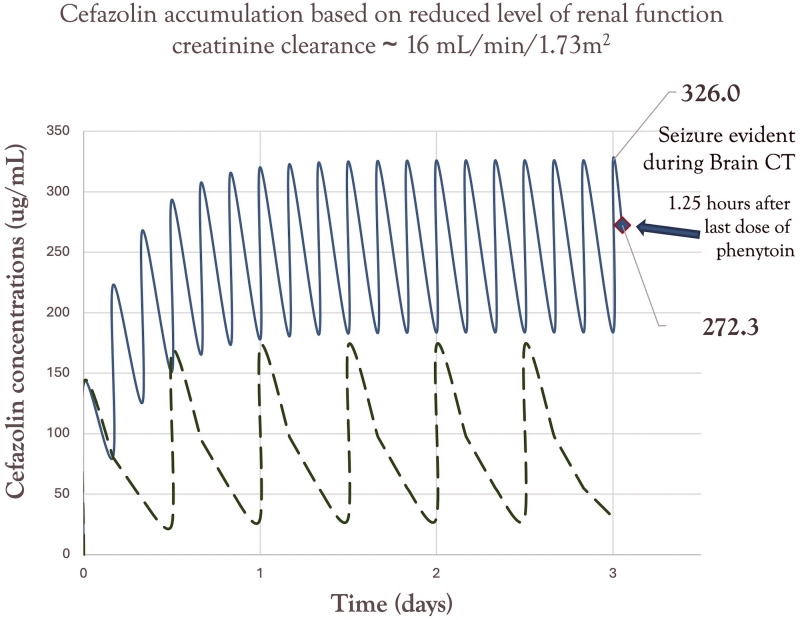
Pharmacokinetic 1-compartment simulation of cefazolin concentration-time profile (dark line) for this 87-year-old, 72.6 kg female, receiving high-dose cefazolin 2 grams i.v. every 4 hours in the presence of reduced renal function (i.e., creatinine clearance of about 16 mL/min/1.73 m^2^). The values of 326 ug/mL and 272.3 ug/mL represent the simulated steady-state peak, as well as the concentration at the time of the seizure, respectively. The dashed line represents the concentration-time profile for this patient with an appropriately dosed, renal function-adjusted regimen of cefazolin 2 grams i.v. every 12 hours. The result was a greater than 3 times drug exposure from her regimen; and therefore, a possible explanation for her seizures by the third day of therapy.

She continued to progressively decline in the low acuity unit while on this supratherapeutic dosing regimen, and because of an ongoing decrease in level of consciousness (LOC) she was transferred to our tertiary-care facility on day 11 of hospitalization. At this point cefazolin was stopped and she was transitioned to cloxacillin 2 g i.v. every 6 hours, however, on review of the electronic record cloxacillin was not started until day 12 of hospitalization. Her sodium, potassium, and calcium remained within normal limits with a slightly elevated thyroid stimulating hormone of 5.6 mU/L and normal T4 of 10.2 pmol/L. An uninfused CT of the brain on day 11 was not suggestive of any acute changes, but immediately thereafter, the patient experienced tonic-clonic movements for 20 seconds followed by eye deviation and tongue biting. Her Glasgow Coma Scale (GCS) score was noted as 3. Phenytoin was loaded at 1000 mg i.v. as an anti-epileptic agent. She had received 4 days of high-dose cefazolin in total. Figure 1 demonstrates the temporal relationship between seizure onset relative to her estimated cefazolin concentration-time profile from the regimen of 2 g i.v. every 4 hours. No drug levels were obtained as this was not an available assay at our site. All graphic outputs were population-based using a 1-compartment pharmacokinetic model based on her body weight of 72.6 kilograms and an average creatinine clearance of 16 mL/min/1.73 m^2^. The distribution space for cefazolin was based on her body weight and the population volume of 0.19 L/kg. The estimated half-life and elimination rates for cefazolin were calculated from the Tozer equation^3^ and using the average fraction of cefazolin renal elimination as 75%. The half-life of elimination was estimated to be as high as 4.8 hours as compared to 1.8 hours for normal renal function in adults.

The impression at that time was that her seizure was related to high-dose cefazolin and exacerbated by her acute renal insufficiency. An electroencephalogram (EEG) performed 24 hours after the event demonstrated no epileptiform activity but did show global slowing consistent with a toxic or metabolic encephalopathy.

Her mentation remained profoundly altered with a GCS score ranging between 7 and 9. She was admitted to the intensive care unit, where she did not regain consciousness and was transitioned to comfort care measures prior to her death. The Naranjo Adverse Drug Reaction Probability Scale score was assessed conservatively as +2 for this patient, which is considered ‘possible’ for this adverse reaction being cefazolin-related. Her seizures were disclosed to her family as a likely an adverse event related to a medication dosing error and a critical incident report was initiated.

## Discussion

Cefazolin is a commonly used beta-lactam antibiotic given its bactericidal nature and generally well tolerated adverse effect profile. Cefazolin is predominantly excreted in the urine with 70-80% as unchanged drug, and requires dosing adjustment for patients with kidney dysfunction.^1^ Supratherapeutic dosing of beta-lactam antibiotics, particularly cephalosporins, has been associated with an increased risk of CNS adverse effects including encephalopathy, seizures, confusion, and coma.^2,3^ There are various risk factors that predispose patients to a higher risk of neurotoxicity including pre-existing CNS conditions, impaired renal function, older age, and the use of higher medication dosing.^3,4^ CNS toxicity can occur between 1-10 days after starting a cephalosporin, with potential resolution 2-7 days following drug discontinuation.^5^

The mechanism of cephalosporin-induced neurotoxicity involves competitive antagonism of the receptor for the inhibitory neurotransmitter gamma-aminobutyric acid (GABA_A_); as well as agonism of the glutamatergic N-methyl-D-Aspartate (NMDA) receptor. A reduction in GABA activity and stimulation of NMDA receptors means less inhibitory and greater excitatory neurotransmission; ultimately leading to neuronal excitation and seizures.^4,6^

Our patient had all of the aforementioned risk factors increasing her predisposition for cephalosporin related neurotoxicity. She also fits within the timeline of toxicity given that her seizures occurred 4 days after being on the supratherapeutic dose of cefazolin. In theory, piperacillin/tazobactam use 48 hours prior to the change to cefazolin could have contributed to neurotoxicity, however, she was on an appropriately dosed regimen of 2.25 grams i.v. every 8 hours and its presence would have been washed out within 24 hours after it was discontinued, which makes this less likely.^5^ Piperacillin/tazobactam could have resulted in elevated serum creatinine as the antibiotic inhibits secretion of creatinine into the urine via blocking anion transporter cells in the tubules.^7^ This has not been shown to affect glomerular filtration rate as measured by cystatin C, but could have acted as a confounder in making her kidney function appear worse with a higher plasma creatinine level.^7^ Even if this was the case her dose of cefazolin was higher than guideline recommended dosing and could have still contributed to a neurotoxicity risk.^7^ Cloxacillin has also been associated with seizures, however, her epileptic activity occurred prior to receiving her first dose of cloxacillin and thus thought not to be a contributor.^8,9^ There were no other significant biochemical abnormalities or medications that would have led to her condition. There is a possibility that her bacteremia and acute illness could have increased her risk of developing septic encephalopathy with seizure and is a potential confounding factor.^10^

The Naranjo Adverse Drug Reaction Probability Scale is a standardized scoring system that helps to assess for causality for adverse drug reactions.^11^ Based on the above information, our patient would score a total of 2 points, which is considered ‘possible’ for this neurotoxicity being related to cefazolin. The scoring criteria in favor of the reaction being related to cefazolin includes +1 for previous conclusive reports on this reaction and +2 for the adverse event appearing after the suspected drug was administered. The scoring criteria against the reaction being related includes −1 for alternative causes that could have caused neurotoxicity on their own with bacteremia and acute illness being on the differential. Unfortunately, Naranjo Adverse Drug Reaction scoring does not give any weight to the possibility for increased risk of adverse reactions secondary to drug accumulation in renal failure, as was her case. Therefore, we think that this case is more likely ‘probably’ drug related. As mentioned, there are limitations to the use of the Naranjo scale in critically ill patients, however, we are unaware of any other tools that are validated for assessing adverse drug reactions in this subset of patients.^11^ Table 1 provides an outline of the Naranjo scale used in this report. Table 1 was adapted from a case report by Murray and Tobias, with the main change in the table being the description of what the various scoring ranges mean.^12^

**Table 1.** Naranjo Adverse Drug Reaction Probability Scale.

Naranjo Score				
Question	Yes	No	Do Not Know or Not Done	Patient Score
Are there previous conclusive reports on this reaction?	+1	0	0	+1
Did the adverse event appear after the suspected drug was given?	+2	−1	0	+2
Did the adverse reaction improve when the drug was discontinued, or a specific antagonist was given?	+1	0	0	0
Did the adverse reaction appear when the drug was readministered?	+2	−1	0	0
Are there alternative causes that could have caused the reaction?	−1	+2	0	−1
Did the reaction reappear when a placebo was given?	−1	+1	0	0
Was the drug detected in any body fluid in toxic concentrations?	+1	0	0	0
Was the reaction more severe when the dose was increased, or less severe when the dose was decreased?	+1	0	0	0
Did the patient have a similar reaction to the same or similar drugs in any previous exposure?	+1	0	0	0
Was the adverse event confirmed by objective evidence?	+1	0	0	0
Total Score				2

Total scores range from −4 to +13; the reaction is considered definite if the score is 9 or higher, probable if 5 to 8, possible if 1 to 4, and doubtful if 0 or less.

Renal failure is one of the most critical risk factors associated with neurotoxicity from beta-lactam antimicrobials. Roger et al have claimed that neurotoxicity is secondary to increased beta-lactam trough concentrations, however, there is no evidence associating whether trough, peak, or area under the curve (AUC) is the strongest correlate for the development of neurotoxicity.^6^ Excessive exposure plays a role, but more research is necessary to identify the best correlated pharmacokinetic parameter.

Chen and colleagues had described a peritoneal dialysis patient with an altered LOC and seizures following the receipt of 1.5 g of cefazolin intraperitoneally every 24 hours for 5 days.^13^ This 76-year-old female patient had a measured cefazolin level of 149.5 ug/mL, which was drawn 16 hours after the night peritoneal dialysis exchange and suggests that the value at the time of the seizures was actually much higher.^13^ This measured concentration associated with a seizure was well below the estimated cefazolin concentration of 272 ug/mL in our patient. This provides further evidence that the level of cefazolin exposure in our patient may have contributed to neurotoxicity.

Upon review of the literature, there are no published studies to our knowledge that have assessed for the toxic effects of cefazolin with a non-traditional dosing regimen of 2 g i.v. every 4 hours. This helps to differentiate our findings from other studies and further outlines the relevance that higher exposure in renal failure contributed to neurotoxicity. Unfortunately, we were unable to obtain a serum drug level of cefazolin as this assay is not available at our site.

Therapeutic drug monitoring (TDM) of beta-lactam concentrations has been a suggested as a strategy to help reduce the risk of beta-lactam neurotoxicity.^6^ Dosing adjustments can be made once steady state concentrations have been achieved, which is often within 24-48 hours of initiation of therapy. Higher empiric doses may be used initially, however, follow-up TDM levels can ensure therapeutic adequacy, while minimizing toxic-range values. The challenges with TDM are the increased costs associated with assaying blood levels, as well as a lack of well-established thresholds for beta-lactam toxicity.^6^ This case report and others are crucial in trying to identify a range in thresholds associated with neurotoxicity, which will be useful for future TDM.

## Conclusion

This case outlines a poor outcome associated with a high and non-traditional dosing of cefazolin for a patient with MSSA bacteremia and concurrent renal dysfunction. High-dose cefazolin may have been a major contributor to the development of CNS ADRs, including seizures and altered mentation. This case serves as a reminder to clinicians for ongoing pharmacovigilance in adhering to treatment guidelines to limit the risk of severe adverse effects when prescribing medications. These factors are critical to ensuring positive clinical outcomes, while minimizing the risk for serious adverse complications. TDM may have a potential future strategy to limit antibiotic overexposure in high-risk patients, but more research must be done to establish appropriate thresholds.
